# Bimanual thumb-index finger indications of noncorresponding extents

**DOI:** 10.3758/s13414-021-02360-8

**Published:** 2021-08-02

**Authors:** Klaus Landwehr

**Affiliations:** Psychologisches Institut, Johannes Gutenberg-Universität Mainz, 55099 Mainz, Germany

**Keywords:** Visual illusions, Euclidean geometry, Psychophysics, Motor control

## Abstract

Two experiments tested a prediction derived from the recent finding that the Oppel-Kundt illusion – the overestimation of a filled extent relative to an empty one – was much attenuated when the empty part of a bipartite row of dots was vertical and the filled part horizontal, suggesting that the Horizontal-vertical illusion – the overestimation of vertical extents relative to horizontal ones – only acted on the empty part of an Oppel-Kundt figure. Observers had to bimanually indicate the sizes of the two parts of an Oppel-Kundt figure, which were arranged one above the other with one part vertical and the other part tilted -45°, 0°, or 45°. Results conformed to the prediction but response bias was greater when observers had been instructed to point to the extents’ endpoints than when instructed to estimate the extents’ lengths, suggesting that different concepts and motor programs had been activated.

## Introduction

The purpose of the present paper is threefold: (1) To provide empirical evidence that humans are able to simultaneously indicate noncorresponding extents by spreading thumb and index finger of their hands accordingly – as postulated by Gibson (1966, pp. 119-120). (2) Using digital indications as an alternative method to measure observers’ performance, to provide a replication of the recent finding, obtained with verbal judgments, that the overestimation of vertical extents relative to horizontal ones only seems to hold for empty extents as opposed to subdivided ones (Landwehr, 2021). (3) To provide a comparison of digital indications and verbal judgments considered as psychophysical research methods. Two experiments, utilizing modified versions of an Oppel-Kundt-type visual-illusion figure, will be reported to illustrate and substantiate the three aims.

Apparently inspired by Katz and MacLeod’s (1949) paper on “The mandible principe in muscular action,” but also by Troland’s (1929) ideas about sensitivity for bodily postures, Gibson (1966) maintained that we were able to indicate linear extents by spreading the thumb and index finger accordingly. This kind of digital or haptic indication could then be signaled to other persons in the service of social communication. Technically, however, due to incessant muscular tremor, it is exceedingly difficult to come up with an unequivocal measure of a person’s intended finger span. For this reason, even when using a movement-tracking system (Landwehr, 2009), I asked experimental participants to transfer their intended indications to a rigid surface. And even so, as noticed by a 5-ms sampling rate on a touch-sensitive computer screen, a somewhat arbitrary decision has to be made on what to use as the subject’s response (Landwehr, 2014, p. 1154). Nevertheless, once a set of criteria is consistently applied, reliable data can be obtained (Landwehr, 2015, 2016).

In the wake of Aglioti et al.’s (1995) influential publication “Size-contrast illusions deceive the eye but not the hand”, manual-size indications have often been used as a “perceptual” measure of observers’ susceptibility to visual illusions, and been compared to manifest grasping (e.g., Daprati & Gentilucci, 1997; Franz, 2003; Haffenden & Goodale, 1998; Westwood et al., 2000). The comparison originally had been motivated by the so-called two visual systems theory, which posits a division of labor between the dorsal and the ventral cortical pathways (Goodale & Milner, 1992; Milner & Goodale, 2006; Ungerleider & Mishkin, 1982). I shall not comment on that theory here,^1^ but study manual indications in their own right. I discuss different methods of analyzing data, and show that comparisons to other forms of motor behavior (notably, grasping) and to verbal judgments are severely limited.

A major theme in psychophysics is the separation of two aspects of observers’ responses: bias and sensitivity. Sensitivity refers to the ability to discriminate stimuli, and bias to a (typically uncorrelated) constant error in responding (e.g., over- or underestimation of linear extents; Macmillan & Creelman, 2005). Illusion researchers traditionally focus on bias, but as noted by Morgan et al. (1990) – and empirically demonstrated with a modified Müller-Lyer illusion figure – sensitivity and bias may coexist with illusion stimuli as well. Different from the standard psychophysical methods, for which metric measures of bias and sensitivity have to be estimated from fitted psychometric functions (Klein, 2001; Urban, 1908), manual indications immediately yield metric data that can be analyzed by regression methods. In a model *y* = *a*δ_*x*_ + *b*, where δ_*x*_ is the difference of the stimulus parameter of interest from the mean of the parameter’s values, the intercept *b* is the observer’s mean response, and so – after subtraction of the mean value of the stimulus parameter – provides a measure of the average over- or undershooting of the target. It is not a measure of bias in the sense of the point of subjective equality, because that point refers to pairs of stimuli that have been compared by observers. For manual indications, the *differences* in over- or undershooting with regard to different targets constitute target- and stimulus-specific measures of bias (Landwehr, 2014). Although the slope *a* of a regression line is related to observers’ sensitivity, it is not comparable to the difference threshold (or just-noticeable difference) as estimated from a psychometric function. Neither is the variable error, which, in linear regression analysis, is customarily determined as the sum of the squared residuals. Regression slopes indicate the degree to which observers were able to reproduce the *intrinsic metric* of stimuli, and variable errors indicate the *consistency* or *precision* with which observers responded. In the results section of the second experiment to be reported, I demonstrate how sensitivity can be evaluated with analyses of variance.

With manual indications, it is even less obvious how to instruct observers to secure meaningful data in the first place. Over the years, I have used a complicated procedure in which haptic signaling was related to manifest grasping, in order to specify precisely which distance between thumb and index finger ought to correspond to a visible extent. Participants were given a short, thin wooden bar, and asked to pick it up by means of a pincer grip with thumb and index finger directed at the bar’s ends. Participants were made aware of the fact that the distance between the digits differed depending on how strong the fingers pressed against the bar. They were told that we were interested in the distance between the digits immediately before the executed grasp because this might be regarded as an estimate of the bar’s length when shown to other people. Finally, participants were made aware of the additional problem that the points of contact of the fingers with the bar did not correspond to the points that would touch the surface to which the estimate was to be transferred. Hence, participants were instructed to decide on a spread between thumb and index finger and then keep this distance until the fingers made contact with the substrate (i.e., the computer screen).

The differences observed by Goodale et al. (1994, Experiment 3) between actual and pretended grasping – differences in trajectory, velocity, duration, and hand aperture – suggest that manual indication probably cannot be used as a substitute to study grasping behavior (see also Westwood et al., 2000; Whitwell et al., 2020). On the other hand, Smeets and Brenner (1999, 2001) have likened grasping to pointing, which suggests that the detour in my original instruction may not be needed. In what follows, I report two haptic experiments with the Oppel-Kundt illusion, which were conducted in strict analogy to a corresponding verbal study (Landwehr, 2021). Experiment 1 used the old instruction, which focused on the indication of lengths, and Experiment 2, which was a replication of the first experiment, used a simplified instruction in which participants were told to try to hit the endpoints of the visible linear extents of a modified Oppel-Kundt figure immediately after the stimulus had been turned off. Note that the new instruction changed the manual-indication task as conceived by Gibson (1966) into a pointing task.

**Fig. 1:**
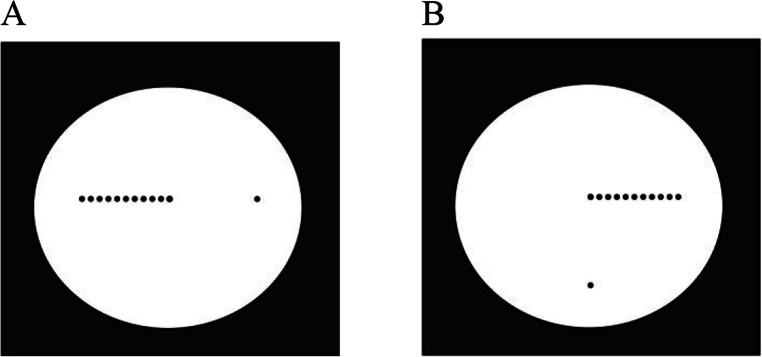
Examples of the Oppel-Kundt-type illusion figures used in Landwehr (2021). *Note*. From “The Oppel-Kundt Illusion and Its Relation to Horizontal-Vertical and Oblique Effects” by K. Landwehr, 2021, *Perception*, *50*(5), pp. 473-474 (10.1177/03010066211006545). Copyright 2021 by Sage Publishing. Reprinted with permission

The Oppel-Kundt illusion (Kundt, 1863; Oppel, 1860-1861) consists of the overestimation of the length of discontinously filled versus otherwise equal, empty extents. Utilizing a variant of Kundt’s original stimulus with one plus 11 dots (Fig. 1a), with verbal responses obtained with the method of constant stimuli, I found the illusion to be significantly greater at a horizontal orientation of the figure as opposed to a vertical orientation (Landwehr, 2021). The attenuation of the illusion was even stronger in a modified figure in which the vertical part was empty and the horizontal part dotted and tilted 90° (Fig. 1b). The effect was interpreted as a stimulus-specific intrusion of the horizontal-vertical illusion (viz., the overestimation of vertical relative to horizontal extents; Fick, 1851), which seems to have acted on the figure’s empty stretch only – making it appear longer, and so work against the usual Oppel-Kundt effect.

**Fig. 2:**
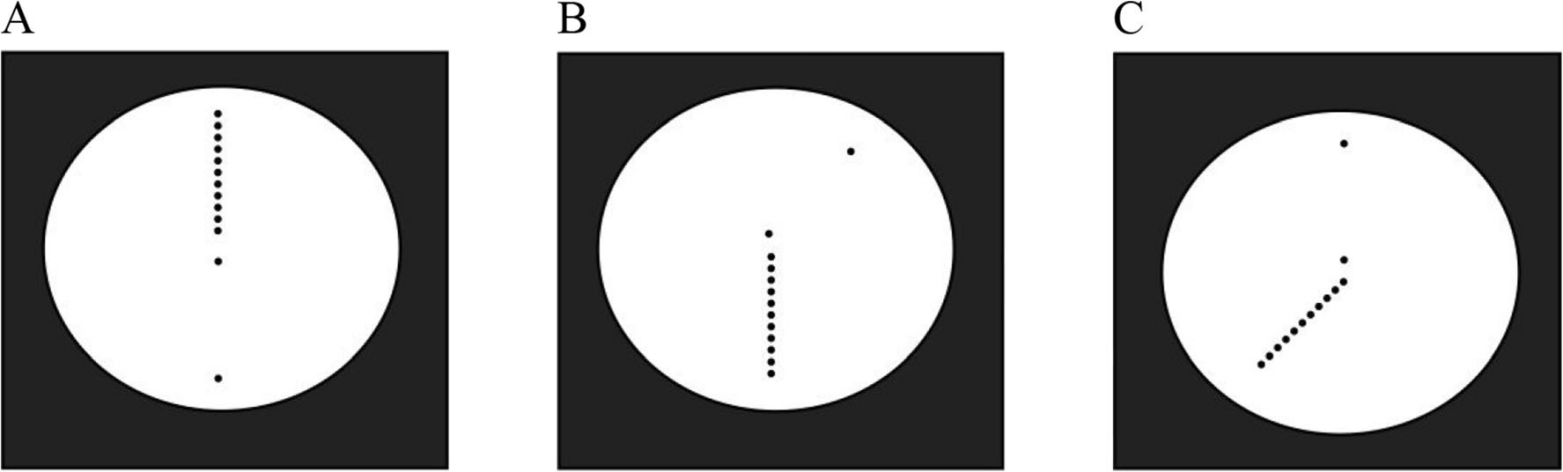
Examples of the modified Oppel-Kundt illusion figures used in the present experiments

As horizontal extents imply awkward arm, wrist, and hand postures for manual indications, haptic analogues of the verbal experiments were prepared for selected stimuli of Landwehr’s (2021) Experiment 2 only. For example, cases like those shown in Fig. 1b had to be left out, so that only vertical Oppel-Kundt figures, and figures in which the two parts of an Oppel-Kundt-type stimulus were vertical or oblique, were used (Fig. 2; for details see the *General method, Stimuli and responses* section).

## General method

### Participants

Twenty psychology undergraduates took part in the experiments, ten per experiment (independent samples). The number of participants had been decided upon a priori in order to achieve statistical power of 1 – β ≥ .95 for repeated-measures analyses of variance (rmANOVAs) with α = β ≤ .05, and *f* ≥ 1 (Faul et al., 2007). With data analyzed by means of paired *t*-tests, larger samples would have been required, but were not available. Results were interpreted only when ex post facto calculations yielded sufficient power. Written, informed consent was obtained from all participants, and they were treated in accordance with the Declaration of Helsinki (World Medical Association, 1964/2013). The department’s ethics committee declared the experiments exempt from obtaining ethical approval because they were bare stimulus-response psychophysics. All participants had normal or corrected-to-normal vision, none was physically handicapped, and all served in partial fulfillment of a class requirement.

### Apparatus

The apparatus used was a duplicate of the one used in Landwehr (2021). Its essential part was a touch-sensitive computer screen (size: 59.6 × 33.5 cm; resolution: 2,560 × 1,440 pixels; response time: 3 ms), which was used for both stimulus presentation and response registration. The screen had been set flush into a table so that the viewing distance to the center of the screen was about 44 cm. Stimuli, drawn from small black dots (diameter: 1.25 mm; visual angle: 1 arcmin), were presented within a circular, light grey window (diameter: 28.5 cm; plane horizontal visual angle: 35.9°; luminance: 228 cd m^-2^; CIE-coordinates: *x* = 0.312; *y* = 0.332; Weber contrast between stimulus and background: C_W_ = - 0.998); the rest of the screen was dark (0.355 cd m^-2^), and there was only faint, indirect illumination of the room.

### Stimuli and responses

There were two types of stimuli: either the filled or the empty part of an Oppel-Kundt-type figure occupied a fixed vertical orientation in the upper or lower half of the computer screen, whereas the complementary part was tilted -45°, 0°, or 45° (Fig. 2). In order to provide separate touch points for the two parts of the figure, a 1-cm gap was introduced between the parts. As in the verbal experiment, five different lengths were used for the two parts of the modified Oppel-Kundt figure – 6, 6.5, 7, 7.5, and 8 cm – and factorially crossed. The cases in which the parts were equally long were deselected but participants were not informed about this. Stimuli were presented for 2 s. Observers were requested to respond as soon as the stimuli were turned off. A temporal window of 3 s was available before the next trial started.

In Experiment 1, participants’ task was to bimanually indicate the lengths of the two parts of the modified Oppel-Kundt figures by simultaneously spreading thumb and index finger of the two hands, and then transferring the indications to the touch-sensitive computer screen, immediately after the stimulus had been turned off. In Experiment 2, the task was to use thumb and index finger of the two hands to point to the endpoints of the two parts of the modified Oppel-Kundt figures, again immediately after the stimulus had been turned off. In view of the apparent simplicity of the tasks, no prescriptions were made about which hand to use for which part of a given figure. Since all figures appeared in mirror- and/or rotation-symmetric forms, it is highly likely that participants used their right and left hands equally often for the filled and the empty extents.

## Experiment 1

For Experiment 1, there were 2 (types of stimuli) × 2 (top-down reversals) × 3 (angular inclinations) × 20 (length combinations) = 240 unique trials. The prediction was that, qualitatively, a similar effect as observed in the corresponding verbal experiment (Landwehr, 2021) would occur: With the filled part vertical and the empty part tilted, the Oppel-Kundt illusion was expected to be in place, but for the inverse configuration, it was expected to be attenuated or even disappear.

### Results and discussion

One participant did not produce a single valid trial and so had to be excluded from the analyses. Data were analyzed with the linear regression method explained in the *Introduction*. Since the stimuli with 0° tilt between the two parts of the Oppel-Kundt figure did not differ for the two types of stimuli as defined in the *Introduction*, data were analyzed separately for these cases versus the cases in which the two parts were tilted -45° or 45° relative to one another. For the vertically oriented stimuli, a paired *t*-test yielded a significant difference between the responses to the empty and the filled parts of the Oppel-Kundt figure, *t*(35) = 4.313, *p* < .000, *d* = 0 .719, 1 - β = .98. On average, the filled part was indicated 0.33 cm longer than the empty part (*SD* = 0.45 cm), corresponding to a per cent amount of illusion of 4.6% (referenced to the mean extent of 7 cm).

For the tilt stimuli, it was important to analyze data separately for the two types of stimuli with either the filled part at a fixed vertical orientation or the empty part in this role. Paired *t*-tests yielded a significant effect in the first case, *t*(35) = 3.821, *p* < .001, *d* = 0 .637, 1 - β = .97, but not in the second one, *t*(35) = 0.199, *p* < .843, *ns*. When the filled part of the modified Oppel-Kundt figure was vertical and the empty part tilted, the filled part on average was indicated 0.70 cm longer than the empty part (*SD* = 1.11 cm), corresponding to a per cent amount of illusion of 10.1%; when the empty part was vertical and the filled part tilted, the filled part was indicated 0.02 cm longer than the empty part (*SD* = 0.65 cm), corresponding to a per cent amount of illusion of 0.3%.

Both sets of findings nicely parallel those of the corresponding verbal study (Landwehr, 2021): To some degree at least, the Oppel-Kundt illusion survived a rotation of the whole figure to a vertical orientation, but the illusion was destroyed when only the empty part of the figure was vertical and the filled part tilted. As before, the most obvious explanation for this pattern of results is an intrusion of the horizontal-vertical illusion (Fick, 1851), which seems to act on the empty stretch of an Oppel-Kundt figure only.

**Fig. 3:**
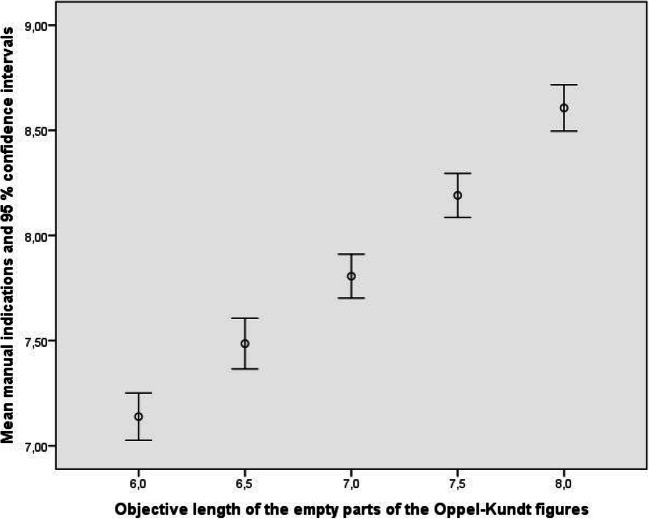
Mean manual indications of the lengths of the empty parts of the Oppel-Kundt-Type figures. *Note*. The data displayed in this and the following figures represent averages, computed across all stimulus conditions and all observers

**Fig. 4:**
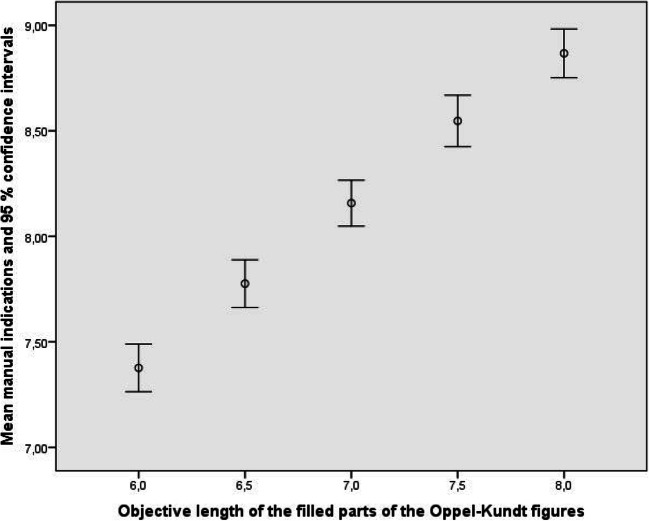
Mean manual indications of the lengths of the filled parts of the Oppel-Kundt-Type figures

Figures 3 and 4 show how well the observers, treated as a group, reproduced the objective sizes of the parts of the Oppel-Kundt figures. The data from the empty parts of the figures can be described by *y* = 0.728 δ_*x*_ + 7.845, R^2^ = .189, and the data from the filled parts by *y* = 0.750 δ_*x*_ + 8.144, R^2^ = .188. Obviously, the observers discriminated the different extents equally well. Since the responses to the different parts of the Oppel-Kundt figures were made simultaneously, and since the to-be-indicated extents were never the same, we can say that the experimental participants bimanually indicated noncorresponding extents *relatively* adequately.

## Experiment 2

Experiment 2 was a strict repetition of Experiment 1 with the pointing instruction substituted for the length-indication instruction. If observers followed the pointing instruction verbatim – namely, to try to hit the endpoints of the linear extents shown – bias might decrease. If, however, observers took the pointing task to be an implicit grasping task, bias might increase because observers would tend to keep a safety margin to be able to execute a grasp rather than to hit the object (Wing et al., 1986).

### Results and discussion

One participant produced only 17 valid trials and so had to be excluded from the analyses. Data were analyzed as in Experiment 1. For the vertically oriented stimuli, a paired *t*-test yielded a significant difference between the responses to the empty and the filled parts of the Oppel-Kundt figure, *t*(35) = 3.723, *p* < .001, *d* = 0 .621, 1 - β = .95. On average, the filled part was indicated 0.54 cm longer than the empty part (*SD* = 0.88 cm), corresponding to a per cent amount of illusion of 7.8%.

For the tilt stimuli, paired *t*-tests yielded a significant effect for the filled part of the modified Oppel-Kundt figure at a fixed vertical orientation, *t*(35) = 6.100, *p* < .000, *d* = 1.017, 1 - β = .99, but not for the empty part in this role, *t*(35) = 0.504, *p* < .617, *ns*. When the filled part of the Oppel-Kundt figure was vertical and the empty part tilted, the filled part on average was indicated 0.88 cm longer than the empty part (*SD* = 0.86 cm), corresponding to a per cent amount of illusion of 12.5%; when the empty part was vertical and the filled part tilted, the filled part was indicated 0.07 cm longer than the empty part (*SD* = 0.88 cm), corresponding to a per cent amount of illusion of 1%.

**Fig. 5:**
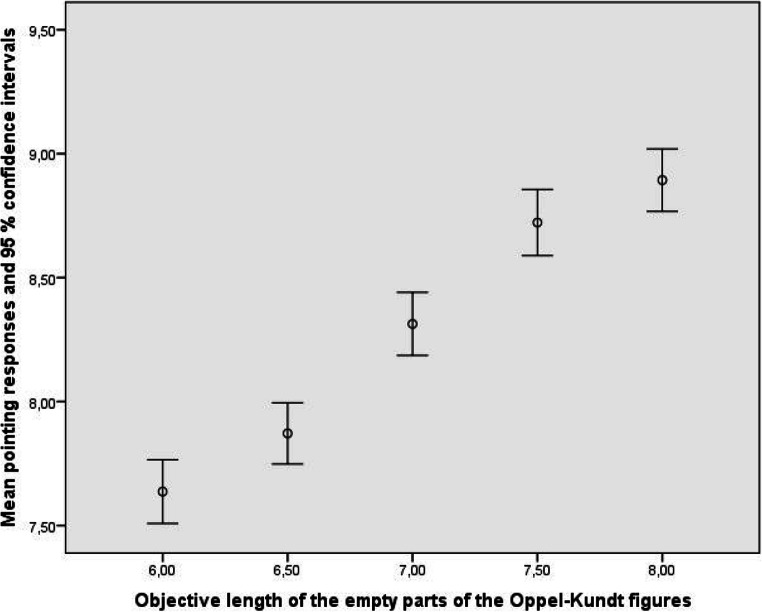
Mean pointing responses to the ends of the empty parts of the Oppel-Kundt-Type figures

**Fig. 6:**
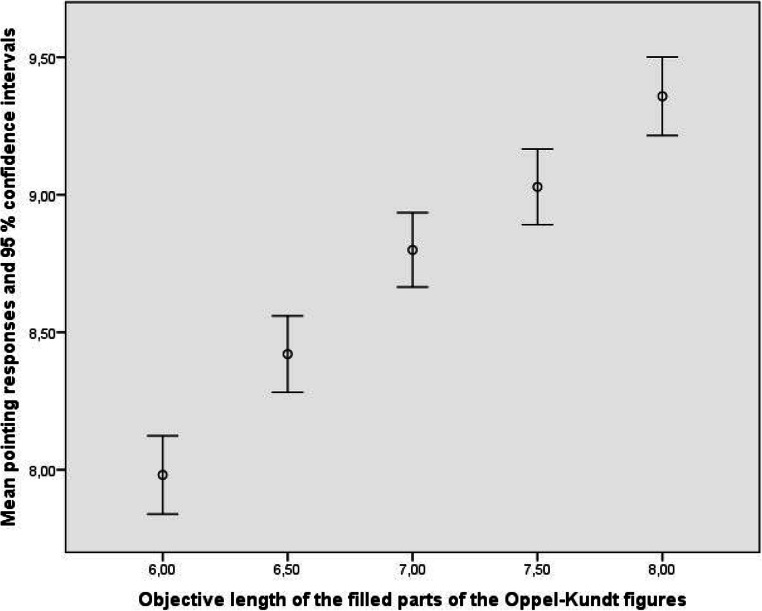
Mean pointing responses to the ends of the filled parts of the Oppel-Kundt-Type figures

Figures 5 and 6 show how well the observers, treated as a group, reproduced the objective sizes of the parts of the Oppel-Kundt figures. The data from the empty parts of the figures can be described by *y* = 0.673 δ_*x*_ + 8.288, R^2^ = .125, and the data from the filled parts by *y* = 0.672 δ_*x*_ + 8.718, R^2^ = .107. Again, observers discriminated the different extents very well, although the discrimination between 7.5 and 8 cm seemed less certain for the empty extents. This was tested by a repeated-measures analysis of variance, and, indeed, the computed contrast was not significant for this case *F*(1, 8) = 2.401, *p* < .160, η_p_^2^ = .231. All other contrasts were significant, with *p* ranging between .000 and .027 and η_p_^2^ ranging between .479 and .861.

Given the similarity in the patterns of the results of Experiments 1 and 2, it seems fair to say that the replication has been successful. However, the different amounts of illusion seen in the two experiments suggest an effect of the different instructions, and, statistically, this was confirmed by paired *t*-tests for both the responses to the empty and the filled parts of the stimuli, *t*(214) = 3.904, *p* < .000, *d* = 0.531, 1 - β = .97, and *t*(214) = 4.887, *p* < .000, *d* = 0.665, 1 - β = .99. Inasmuch as bias increased and discriminative performance slightly worsened, it seems possible that the observers in Experiment 2 indeed took the pointing task to be a grasping task. This supposition can further be tested by comparing one-finger pointing to isolated dots with two-finger pointing to two dots or to the endpoints of a continuous line.

## General discussion

As before (Landwehr, 2009, et passim), manual size indication has proven a useful method to study visual perception with the aim of separating observers’ sensitivity and response bias. Utilizing an Oppel-Kundt-type visual-illusion figure, it was shown that observers were quite proficient in discriminating the lengths of the two parts of such a figure, while at the same time falling prey to the illusion. More specifically, the Oppel-Kundt illusion (viz., the overestimation of the length of its filled part as compared to its empty part) still occurred at a vertical orientation of the stimulus, but it was destroyed when the empty part of the figure was vertical and the filled part tilted to an oblique orientation – but not in inverse configurations. As such, the presently reported results from two haptic experiments provide a twofold confirmation of a finding first reported from a verbal experiment (Landwehr, 2021): The horizontal-vertical illusion (viz., the overestimation of vertical extents relative to horizontal ones) does not appear to hold for a textured linear extent. The generality of this theorem requires further research with different kinds of filler motifs.

How do these results compare with the work of others? Previously, bimanual length indications have not often been used, and, when used with illusion figures, data were analyzed with respect to bias only. Vishton and Fabre (2003) found uni- and bimanual size indications to be the same – and refractory to the Ebbinghaus illusion^2^ – when executed out of view beneath the surface of a table on which the illusion figure had been displayed, but they found unimanual indications to be significantly biased when the executing hand was in view. The authors noted two things, both of which may or may not be true: (1) A post-experimental interrogation suggested "that the two-handed manual estimation task was accomplished by making two sequential one-handed estimations rather than a single, simultaneous judgment" (p. 386), and (2) "Having the hand in view [allowed for] a direct visual comparison" (p. 389). In the procedure that I used in my present experiments, participants were forced to move their extremities simultaneously,^3^ and due to the imposed temporal constraint, there was little chance to visually compare one's digital indications with the current calibration of the stimulus – which comparisons would also have been compromised by perspective distortions. It rather seems that, in both Vishton and Fabre's first experiment as well as in mine, observers performed a cross-modal matching between vision and haptics, and that the unrestrained vision of one's hand in Vishton and Fabre's third experiment – the hand being placed side by side with the stimulus – distracted from the task and so boosted bias.

Dewar and Carey (2006) observed biased bimanual indications (which they indeed called matchings) with a frontoparallel Müller-Lyer display.^4^ The authors also had imposed a 3-s temporal window for responding, and instructed participants to act on the two parts of the stimulus simultaneously. However, different from my experiments, "Participants were not allowed to align their hands with the stimulus array. They were free to observe both the stimulus and their hands during [the] task" (p. 1503). Yet, given the vertical orientation of the stimulus – whereas the matching task had to be performed on a horizontal table surface – it is difficult to see how the concurrent viewing of one's hands and the stimulus could have helped in solving the task. Again, looking back and forth between the stimulus and one's hands may have distracted from the task.

Foster et al. (2012) replicated Dewar and Carey’s (2006) findings, but found no difference in the amount of illusion under closed- versus open-loop conditions (i.e., with the hands in or out of view). As the authors argue, "This is unsurprising, as participants should not benefit much from vision of hand and stimuli in the closed loop [manual estimation] condition, as they never view their hands approaching the object" (p. 3396). If sound,^5^ the argument entails that my procedure should yield less bias than other closed-loop procedures. Since different illusion figures have been used, a test of this conjecture requires a repetition of my present experiments with a Müller-Lyer display. However, Foster et al.’s (2012) finding is at odds with the results of Vishton and Fabre’s (2003) first experiment (not cited by Foster et al., 2012), so that another replication, also using the same illusion figure (Müller-Lyer or Ebbinghaus), and the same *or different* means to occlude vision of one's hands, is needed.

In the discussion of their work, Foster et al. (2012) drew attention to the possibility of an interaction between the activities of the two hands: When two objects of different size have to be responded to simultaneously, an averaging *or* a contrasting effect might occur, and Foster et al. asserted having obtained some evidence for the latter type of effect for manual indications but not for grasping. However, if, in a factorial design, responses to different targets are plotted against opposing target sizes, there will always be a negative slope (or correlation) because larger target size_1_ will in general go with smaller target size_2_. For my own data, plotting and analyzing responses separately for the different target sizes used revealed no significant effects of the opposite target sizes (*p*s ranging between .076 and .960); apparently, as postulated by Smeets and Brenner (1999, 2001), observers operated their hands and fingers independently from each other.

Although the Oppel-Kundt illusion continues to be much researched (e.g., Mikellidou & Thompson, 2014), most often, a “ticks” figure has been used, so that results may not compare well with those that I obtained with a “dots” figure. More seriously, only a minimum of variations has been tried, mainly concerning the number and size of the dividers of the figure’s filled part (e.g., Spiegel, 1937; Wackermann & Kastner, 2009), and the whole figure has almost always been presented in horizontal orientation. Only Bulatov and Bertulis (1999); Bertulis & Bulatov, 2005) used a figure that had parts that could be bent at a variable angle. These authors also used a dots figure, which is immune to rotations. A ten-dots filled part was used as standard, which was fixed at one of four orientations (0°, 90°, 180°, and 270°), and observers had to adjust the outer dot of the empty part, which was rotated full circle in steps of 7°, so that the two extents appeared to be the same. A laterality effect emerged: For the horizontal orientations of the standard, there was no illusion, when the test was aligned to the right of the standard, and for the vertical orientations of the standard, there was no illusion when the test was at right angles to the left of the standard. These null effects may have been due to the observation conditions; observers viewed the stimuli monocularly with their right eye only. A replication is needed with observers using their left eye instead. Either way, the findings – partly at least – accord with mine: The Oppel-Kundt illusion mainly seems to apply to horizontal extents, and for L-type configurations, when the filled part of the figure is vertical, the horizontal-vertical illusion seems to be ineffective, so that the whole illusion tends to be attenuated or even annihilated.

Amounts of illusion differed considerably between studies but do not seem to be linked to the kinds of stimuli and the psychophysical methods employed. Looking only at the generally used horizontal figures, we find that Bulatov and Bertulis (1999) observed an overestimation of the filled part of a dotted Oppel-Kundt figure with the empty test part to the left of the filled reference part by about 14%. Wackermann and Kastner (2009) obtained a similar value with a homogeneous ticks figure. Both groups of authors had used an adjustment procedure. Mikellidou and Thompson (2014), however, while also using ticks figures but the method of constant stimuli, obtained much smaller values (around 5%). Also utilizing the method of constant stimuli but a dots figure, I observed 13% (Landwehr, 2021). A procedural variable that might be responsible for the differences is viewing time, which was unlimited in Bulatov and Bertulis’s (1999) study, 2 s in Landwehr’s (2021), but only 750 and 1,000 ms in Mikellidou and Thompson’s (2014) experiments.

The second major finding of the presently reported research was that the two experiments, which used the same dependent measure (separation between thumb and index finger when touching the computer screen), yielded different amounts of bias. The different instructions used – and the different tasks posed – do not seem to have been differently difficult because, on average, observers did not differ much with regard to discriminative sensitivity. It rather seems that the different instructions induced different attitudes in the participants. In Euclidean geometry, the shortest possible continuous path from one point to another point and the air-line distance between two discrete points are equivalent. Perceptually and behaviorally, however, this need not be so. In fact, line and empty space versions of the Oppel-Kundt illusion (Robinson, 1972) are prototype examples of this psychological nonequivalence. Westheimer and McKee (1977) found difference thresholds for the endpoints of individual lines to be twice as high as those for two equidistant, isolated dots, suggesting that both sensitivity and bias can be affected (as they were, to some degree, in Experiment 2). On the assumption that the subjects of the presently reported experiments stuck to the instructions, they will have conceived their task in terms of linear extents to be estimated in terms of length (or distance) in Experiment 1, but in terms of positions of isolated points in Experiment 2 – possibly without any reference to the concept of distance at all (cf. Mack et al., 1985, for the possibility of a dissociation of position and extent within a figure). A *mixed* task, in which observers are requested to provide manual indications of length *and* to hit the endpoints of given extents as precisely as possible, might temper the excessive response bias seen in Experiment 2 down to the level seen in Experiment 1.

Throughout this paper, I have been emphasizing the differences between manual indications and verbal judgments, considered as psychophysical methods to measure sensitivity and bias in observers’ responses. Table 1 summarizes these points and adds a few thoughts on where the differences might come from. The methods are compared with regard to the perceptual modalities involved, the responses that observers are required to provide, the kinds of data that these responses yield, and the methods with which data can be analyzed. The responses are further analyzed with regard to their mathematical properties and with regard to possible ways in which they are generated. Similarly, the data are also characterized mathematically and with regard to their empirical preponderance.

**Table 1:** Comparison between visually guided verbal-judgment tasks and manual indications (haptic signaling)

Task	Perceptual modality	Response		Data		Analysis
		Type	Generation	Type	Empirically observed	
Ordinal verbal judgment	Vision	Discrete (most often: binary)	Decision	Frequencies	Probabilistic (most often: cumulative Gaussians)	Psychometric functions
Metric haptic indication or reproduction	Vision+haptics	Continuous	Imitation or aiming	Ratio scale	Deterministic (most often: linear)	Regression or ANOVA

*Note*. An adjustment task is intermediate between haptic reproduction and verbal judgment in so far as it contains a motor component but also a final decision about the appropriateness of the setting

Ordinal verbal judgments are typically based on visual stimuli, but other modalities also apply (e.g., audition). Such judgments imply a comparison between stimuli or stimulus elements – in the present case: the sizes of linear extents – and different heuristics may be tried, for example, imaginary translation, rotation, reflection, or glide reflection of the to-be-compared extents to establish their mutual congruence or incongruence, the application of an internal standard, or the use of the mean of the stimuli as a reference (cf. Morgan et al., 2000, who deliberately forced experimental participants to revert to such a strategy). Since, in experiments, verbal responses have to be sorted into discrete categories – here: into *shorter* versus *longer* – they will ideally be based on deliberate decisions (Tanner Jr. & Swets, 1954). This is most obvious in forced-choice procedures, but it also holds for same-different and oddity designs (Macmillan & Creelman, 2005). If the stimuli to be compared are very similar, observers will suffer from uncertainty. This state of affairs typically yields a frequency distribution that can best be fitted by a probabilistic function (ibid.).

Manual indications or reproductions are continuous and so, when digitized, are likely to yield linear, ratio-scaled data, that is, data with a true zero and equal-interval steps (Stevens, 1946; Teghtsoonian & Teghtsoonian, 1965). These data usually show a linear trend, except when the size of the indicated object exceeds the maximum possible finger span (Chan et al., 1990). Responses may be based on aiming (as in the present Experiment 2) or on imitation (as in the present Experiment 1). As for the latter, due to the temporal constraints imposed in my experiments, observers, although they can see their hands, hardly have time to compare their finger spans to the stimuli. Also, due to the different perspectives under which stimuli and finger spans are seen, this would be of little help. Still, observers, inasmuch as they are probably accustomed to the practice of haptic signaling from various everyday activities, may perform an imaginary comparison between the stimulus and their thumb-index finger spreads. As suggested in the discussion of Vishton and Fabre’s (2003) first experiment, this will most likely be a visual-haptic cross-modal matching. Concerning the pointing task, it seems more likely that observers do not engage in any kind of comparison at all. As suggested in the discussion of Experiment 2, observers seem to perform two-finger pointing movements as vicarious grasping. Hence, manual indications, if based on imitation, may contain a cognitive component, but if based on pointing, display a greater affinity to plain motor behavior, or direct stimulus-response coupling (cf. Gibson, 1950, p. 69, for the notion of “immediate processes”).

## Data Availability

All data generated and analyzed during the current experiments are available from the author on reasonable request.

## Footnotes

1 Interested readers are referred to Kravitz et al. (2011, 2013) for indepth neurophysiological state-of-the-art reviews, to Kopiske et al. (2017) versus Whitwell and Goodale (2017) for an exchange of opinions about the implications of psychological experiments and case studies, and to Smeets and Brenner (2006) for an early critique that the whole body of research generated in this tradition may have been misconceived right from its inception. The debate has recently been continued in a special issue of the journal *Cortex* (2018, *98* [January]).

2 The original Ebbinghaus illusion (Wundt, 1898) consists of the overestimation of the size or the diameter of a circle that is surrounded by other, small circles, as compared to a circle of equal size or diameter that is surrounded by larger circles.

3 Foster et al. (2012) measured movement times of the two hands and found them to be nearly identical, suggesting that, if pressed, observers are able to respond simultaneously.

4 The Müller-Lyer illusion figure consists of two, typically aligned, parallel lines of equal length, at the ends of which inward- or outward-pointing arrowheads have been attached; usually, the line with inward-pointing heads is seen as longer (Müller-Lyer, 1889).

5 Santello and Soechting (1997) varied the shape, size, distance, and orientation of solid objects (cubes, parallelepipeds, and cylinders) but, under open-loop conditions, found no effects of the latter three variables on digital indications when index, middle, or ring finger and thumb were used, suggesting that neither the distance between hand and object nor alignment of finger span and face of a 3D object are necessary for correct indications.
